# Efficacy of Different Root Canal Irrigants on Smear Layer Removal after Post Space Preparation: A Scanning Electron Microscopy Evaluation

**DOI:** 10.22037/iej.2017.36

**Published:** 2017

**Authors:** Rahele Mirseifinejad, Mehdi Tabrizizade, Abdolrahim Davari, Fateme Mehravar

**Affiliations:** a *Operative Dentistry Department, Dental School, University of Shahid Sadughi, Yazd, Iran; *; b * Department of Endodontics, Dental School, University of Shahid Sadughi, Yazd, Iran; *; c * Student, Dental School, University of Shahid Sadughi Yazd, Yazd, Iran*

**Keywords:** Post Space, Root Canal Irrigant, Scanning Electron Microscopy, Smear Layer

## Abstract

**Introduction:**

Effective durable adhesion between post material and dentine using resin cements is essential for longevity of restoration. The aim of this *in vitro* study was to compare the effect of different irrigants on smear layer removal after post space preparation.

**Methods and Materials:**

A total of 75 extracted anterior human teeth were selected. The canals were instrumented by rotary system and then were filled. After preparing the post space, teeth were divided into 5 groups according to irrigants: 17% EDTA; 17% EDTA+2% CHX; 5.25% NaOCl; 17% EDTA+5.25% NaOCl; and saline. The canals were irrigated with 5 cc of each irrigants for 1 min. Specimens were examined with scanning electron microscopy (SEM). Hulsmann’s score was used for marking of smear layer removal at coronal, middle and apical thirds of post space. The data were analyzed using the Kruskal-Wallis and Mann-Whitney U tests.

**Results:**

The results revealed that subsequent use of 17% EDTA+5.25% NaOCl was more effective than the other groups in smear layer removal. No statistical difference was found among different levels of root canal within each group.

**Conclusion:**

It can be concluded that 17% EDTA+5.25% NaOCl could be an effective irrigant for smear layer removal after post space preparation.

## Introduction

Restorative dentistry has improved by providing the adhesion of dental materials to mineralized tooth structure [1]. Loss of large amount of coronal tooth structure necessitates the use of intra-canal posts for increasing retention of coronal restoration [2]. To improve tooth longevity, fiber posts are effective in restoring endodontically treated teeth with loss of large coronal structure and their combined use with resin cements and restorative materials can create a structural-functional complex with root dentine [3].

Therefore, effective adhesion in post-cement and cement-dentine interfaces is an essential part of restoration longevity [4]. 

Many *in vitro* studies have been performed on evaluation of different adhesive systems and also post and dentine pretreatments for increasing the bond strength, and have shown that the cause of most failures is the bond failure between post and dentine [3]. Another study has stated that debonding of resin and dentine interface in consequence of dentine hybridization´s problems is the cause of the most common post failures [2]. Therefore, achieving an effective bonding to root canal walls is challenging, according to undesirable geometry of root canal and limitation of adhesive´s physical properties [4]. In addition, root dentine treatments during root canal therapy might interfere with adhesion to the root dentine [4]. 

Smear layer is an amorphous irregular layer on the root canal walls formed during biomechanical preparation of root canal [5] and also post space [6]. It includes a superficial 1 to 5 μ-thick layer with a weak bonding to dentine structure and 40-μ smear plugs packed inside the dentinal tubules [7]. Presence of smear layer may increase the possibility of micro flora and toxin presence in canal space and decrease the favorable seal and also it may prevent penetration of intra canal medicine into the dentinal tubules [5, 8]. In addition, dentine hybridization can be affected by some factors like: irrigants, dentinal tubule obstructions during instrumentation, post space preparation and the type of adhesive systems [2].

There are many contradictions in smear layer removal from root canal walls [9]. Many investigators believe that smear layer should be removed from dentinal canal walls, not only because it can contribute to survival and reproduction of bacteria, but also, it might lead to their re accession to dentinal tubules and reinfection of canal space [7]. Previous studies have shown that smear layer removal can open the occluded dentinal tubules more easily and improve the adhesion of post to the root canal walls [4]. On the other hand it is stated that smear layer could prevent bacterial invasion to dentinal tubules due to its barrier role against bacterial metabolites. Dentine bonding of adhesive materials to root canal walls depends on demineralized surface hybridization [10] and resin tag formation [11]. Application of adhesives without removing the smear layer can cause hybridization of this layer with weak bonding interface [11]. Therefore, dentinal adhesion effectiveness mostly relies on smear layer removal and resin-dentine interface formation [12]. A systematic review by Violich *et al*. [13] have concluded that removing the smear layer contributes to disinfected root canal space and eventually leads to improved adaptation of filling materials to root canal walls.

Chemical irrigant solutions play an essential role in chemo mechanical preparation of canal space through removal of pulpal and bacterial remnants from the root canal [4]. However, not a single solution could dissolve both organic and inorganic components of smear layer [5]. New methods for smear layer removal include using of chelator agents during root canal treatment or post space preparation whether alone or as a final irrigant in combination with other solutions which are tissue dissolvents [5]. The ability to dissolve both organic and mineralized (inorganic) tissues, antimicrobial effect and compatibility with the periapical tissues are some of desirable properties of root canal irrigants [14].

Several studies evaluated the effect of different irrigants in root canal treatment procedure but there are few studies about smear layer removal after post space preparation in different root levels and there are conflictions.

Sodium hypochlorite (NaOCl) is one of the most common irrigation solutions in root treatment [14]. It has strong antimicrobial effect and it is able to dissolve necrotic and organic tissues, however it is not capable of dissolving inorganic components of smear layer [15]. It may also dissolve vital tissues in high concentration [1, 4].

Chlorhexidine gluconate (CHX) is also an irrigation solution that is desired for its antimicrobial effect [16] and tissue compatibility [1]. CHX does not interfere with dentinal matrix collagen, therefore quality of dentine layers is preserved [2].

Ethylenediamine tetra acetic acid (EDTA) is a chelating agent that can dissolve the mineralized part of smear layer [16]. Although the main function of EDTA is elimination of smear layer, but dentine erosion may happen with exposure of more than 10 min [4].

According to the few number of studies on the effect of irrigation solutions on smear layer removal after post space preparation the aim of this *in vitro* study was to compare the effect of different irrigation solutions on smear layer removal from root dentine after post space preparation by scanning electron microscopy (SEM).

## Materials and Methods

A total of 75 extracted anterior human teeth with single and straight canals were selected for this study and stored in normal saline 5 months after extraction. Teeth were intact with closed apices and no signs of resorption. The average length of roots were about 13 mm. The access cavity was prepared in each tooth and canal patency was done with a #10 or 15 K-file (Diadent, Burnaby, BC, Canada). Preparation of root canals was performed using ProFile instruments (Maillefer, Ballaigues, Switzerland) installed on a gear reduction handpiece (Sirona Dental Systems GmbH, Bensheim, Germany) powered by a torque-controlled motor (Silver; VDW GmbH, Munich, Germany). Canals were prepared using crown down technique, under constant irrigation with 5 mL of normal saline between files. Obturation of prepared root canals was performed by using standard gutta-percha cones (Diadent Group International Inc., Chongju, Korea) and AH-26 sealer (Dentsply, Tulsa Dental, Tulsa, OK, USA) using lateral condensation technique. After storing the teeth at 37˚C and 100% humidity for 1 week, the anatomic crown of each tooth was cut from cementoenamel junction. Then the gutta-percha was removed and a post space was prepared with low speed Peezo drills (Mani, Tochigi, Japan). Drilling continued until at least 4 mm of the root fillings was remained at the apical level to ensure apical seal preservation. Confirmation radiographies were taken.

All the teeth then were randomly assigned to the following 5 groups: Group 1, 5 mL of normal saline; group 2, 5 mL of 5.25% NaOCl; group 3, 5 mL of 17% EDTA followed by 5 mL of 5.25% NaOCl; group 4, 5 mL of 17% EDTA; group 5; 5 mL of 17% EDTA followed by 5 mL of 2% CHX. Irrigation continued for 60 sec for each irrigant. The irrigants activity was ceased by using 2 mL of normal saline for 1 min.

The root canals in each group were dried with multiple paper points. Each tooth was then split longitudinally in the buccolingual direction using a diamond disc. One of the halves was selected and examined under SEM (Vega II XMU, Tescan, Czech Republic) at the coronal, middle and apical levels of post space. This SEM method did not need any pretreatment. The numbers of dentinal tubules opening were observed under ×1000 magnification at each level for each tooth and were marked from 1 to 4 according to the method suggested by Hulsmann: *score 1*, all the dentinal tubules are completely open; *score 2*, more than 50% of dentinal tubules are open; score 3, less than 50% of dentinal tubules are open; *score 4*, near all of dentinal tubules are covered by smear layer [17].

Score of open dentinal tubules at each level were evaluated by 2 blinded examiners. The least correlation coefficient between two observers was 96% based on Friedman correlation test. The final scores were statistically analyzed with the Kruskal-Wallis and Mann-Whitney U tests using SPSS software (version 18.0, SPSS, Chicago, IL, USA). The significance level was set at 0.05.

## Results

In control group, a thick smear layer (*score 4*) and more blocked tubules was observed in more samples compared to other groups. The most number of samples with complete smear layer removal was observed in 17% EDTA + 5.25% NaOCl group (totally in 9 surfaces). More and larger dentinal tubules were visible in coronal and middle thirds.

In other groups (17% EDTA, 17% EDTA + 5.25% NaOCl and 5.25%NaOCl) smear layer was removed partially in most cases especially in coronal and middle thirds compared to apical third.

The data on smear layer removal scores for each group at 3 levels of post space surfaces are presented in Table 1. According to *P*-values, no significant difference was found between different parts of post space levels within one experimental group (*P*>0.05), except 17% EDTA + 2% CHX group (*P*<0.05).

Significant difference was found among different groups at coronal third (*P*=0.001) and also in middle and apical thirds (*P*=0.000).

When experimental groups were compared statistical analyses in Mann-Whitney U test revealed significant differences between EDTA + NaOCl group and EDTA and also between EDTA + NaOCl group and saline samples, in coronal third (*P*<0.05). 

Statistical difference was found between samples in saline groups and NaOCl and saline and EDTA + NaOCl samples, in middle third. The same difference was found between groups EDTA + NaOCl and EDTA, EDTA + NaOCl, EDTA + CHX and EDTA + NaOCl and saline in apical third. There were no significant differences among other groups in each level. 

## Discussion

Preparing post space in endodontically treated teeth requires gutta-percha and sealer removal which leads to deposition of smear layer and debris on root canal walls and as a consequence, obstruction of dentinal tubules is likely [11]. It seems to be desirable to remove smear layer as it increases the dentine permeability [17]. Adequate adhesiveness of fiber posts and resin luting systems to root canal walls is based on micromechanical retention made by demineralized radicular dentine surface and resin tag formation [12]. Therefore, using adhesive systems without smear layer removal decreases the bonding of adhesives to canal walls because of weak attachment of smear layer to dentine [11]. A critical step for optimal post retention after preparing the post space is cleaning the dentinal surface of canal walls [4]. Some studies have been performed to evaluate the effect of different irrigants for smear layer removal [11]. Until now, no single irrigation solution can dissolve both organic and inorganic components of smear layer. Removing smear layer and debris is the important goal of irrigation [11]. A number of irrigants that have been studied before and their efficacy have been proved including 17% EDTA and 5.25% NaOCl [12]. Moreover, 2% CHX is also recommended for root canal therapy because of its antimicrobial effect and biocompatibility [2].

**Table 1:** Smear layer removal scores for each experimental group on root level

	**Score N (%)**					***P*** **-value**
		**1**	**2**	**3**	**4**	
**17% EDTA**	Coronal	2 (13.3)	4 (26.7)	3 (20.0)	6 (40.0)	0.861
	Middle	1 (6.7)	4 (26.7)	6 (40.0)	4 (26.7)	
	Apical	0 (0)	4 (26.7)	6 (40.0)	5 (33.3)	
**17%EDTA+2% CHX**	Coronal	2 (13.3)	5 (33.3)	4 (26.7)	4 (26.7)	0.021
	Middle	0 (0)	5 (33.3)	8 (53.3)	2 (13.3)	
	Apical	0 (0)	0 (0)	8 (53.3)	7 (46.7)	
**5.25% NaOCl**	Coronal	1 (6.7)	10 (66.7)	3 (20.0)	1 (6.7)	0.275
	Middle	2 (13.3)	6 (40.0)	5 (33.3)	2 (13.3)	
	Apical	1 (6.7)	5 (33.3)	6 (40.0)	3 (20.0)	
**17% EDTA+ 5.25% NaOCl**	Coronal	7 (46.7)	5 (33.3)	3 (20.0)	0 (0)	0.100
	Middle	2 (13.3)	8 (53.3)	5 (33.3)	0 (0)	
	Apical	0 (0)	12 (80.0)	3 (20.0)	0 (0)	
**Saline**	Coronal	0 (0)	2 (13.3)	8 (53.3)	5 (33.3)	0.396
	Middle	0 (0)	0 (0)	7 (46.7)	8 (53.3)	
	Apical	0 (0)	4 (26.7)	4 (26.7)	7 (46.7)	

**Figure 1:**
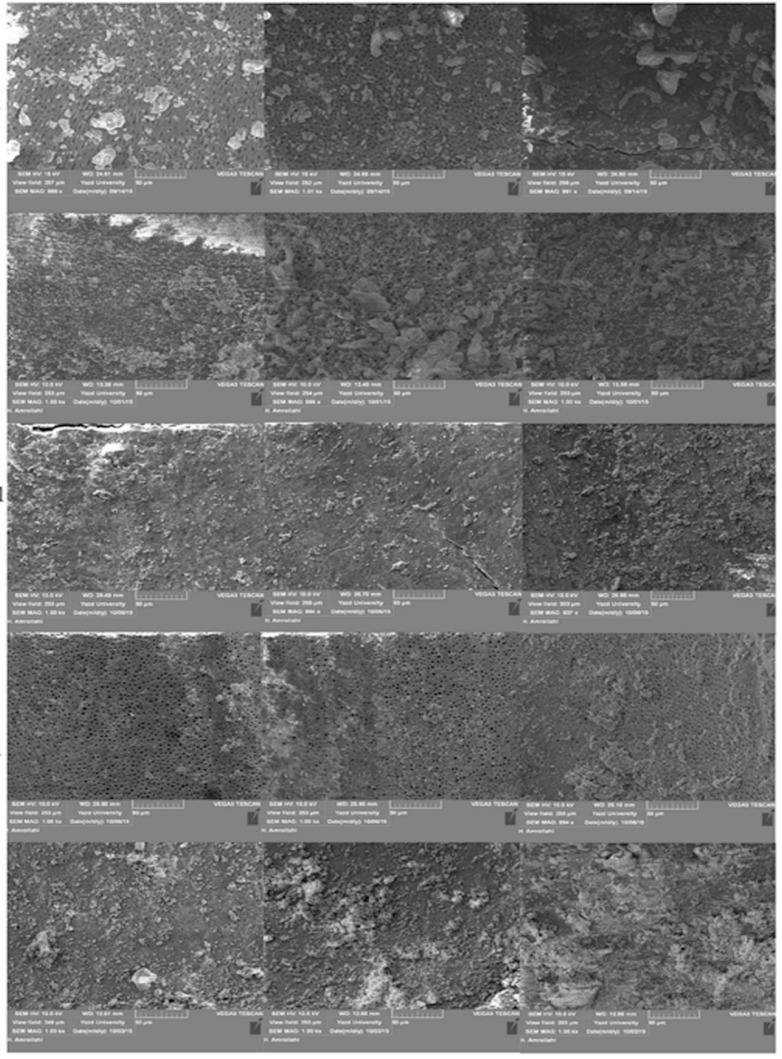
Amount of smear layer removal and open dentinal tubules after irrigation with experimental solutions in coronal, middle and apical levels (left to right

The result of current study showed that irrigating the post space with 17% EDTA followed by 5.25% NaOCl could be more effective in smear layer removal in coronal, middle and apical thirds of post space in comparison with other groups. Ankurda *et al*. [8]and Arisu *et al*. [6] showed that irrigating the post space [6] or root canal [8] with 17% EDTA + 5.25%NaOCl could significantly improve the smear layer removal efficacy. EDTA is a chelating agent and leads to dentine demineralization and leaves the collagen scaffold exposed. Irrigating this exposed surface with 5.25% NaOCl causes the collagen dissolution [6]. Therefore, these two irrigants could have an effect on organic and inorganic components of smear layer amorphous particles. On the SEM images of 5.25% NaOCl samples, there were more open dentinal tubules. Irrigating post space with each one of irrigants lasted for 1 min. Using 17% EDTA more than 1 min may lead to dentinal erosion [11]. Zhang *et al. *[18] also showed that 17% EDTA + 5.25% NaOCl had a considerable effect on the cleaning of post space surface.

In this study, 17% EDTA, 5.25% NaOCl and 17% EDTA + 2% CHX showed statistically similar results (*P*>0.05). On the other hand, 17% EDTA and 17% EDTA + 2% CHX in coronal and apical thirds and 5.25% NaOCl just in apical third showed no significant differences compared to control group (saline) (*P*>0.05). In none of the three mentioned groups smear layer was eliminated completely. However, according to SEM images, and compared to control group, smear layer was removed to some extent. Andrabi *et al.* [19] reported that 17% EDTA were more effective on smear layer removal in each level compared to control group and this is in agreement with the result of the current study.

Also, Andrabi *et al. *[19], Gu *et al. *[12] and Elnaghy *et al.* [4] concluded that 17% EDTA is significantly more effective on smear layer removal which is in contradiction with our findings. Maybe this disagreement is derived from the structural difference between the dentinal wall and smear layer. Unlike the dentine, smear layer does not have a well-organized structure and its organic and inorganic components have an amorphous and irregular structure, so 5.25% NaOCl can be effective in the first step and collagen fibers are not protected with hydroxyapatite crystalline. Takeda *et al.* [20] stated that EDTA is not able to completely remove smear layer explained this as a result of decreasing pH during demineralization and self-limitation effect. Zand *et al.* [21] compared smear layer removal efficacy of NaOCl, EDTA and an experimental irrigant containing Papain, EDTA, tween 80 and CHX and they did not find out any differences among the groups. In another study Andrabi *et al.* [19] compared EDTA, Smear Clear, BioPure MTAD and NaOCl after final endodontic preparation. They found no difference among the groups in coronal and middle part of the canal but in apical zone, BioPure MTAD was more effective than others. 

Elnaghy *et al.* [4] also reported that 17% EDTA and 17% EDTA+2% CHX showed no significant difference in smear layer removal and opening of dentinal tubules. That is in agreement with current study but these two irrigants showed to be more effective compared to 5.25% NaOCl and the control group and this is in contradiction too. In current study, the only significant difference between 5.25% NaOCl and 17% EDTA + 2% CHX was noticed in apical third and 5.25% NaOCl had better effect. Irrigation with 17% EDTA + 2% CHX as a final irrigant in the study by Tuncer *et al.* [15], showed more effective smear layer removal in coronal and middle third compared to 5.25% NaOCl and this is a confliction, too. According to our findings it seems that 2% CHX has no effect on smear layer removal.

Different results of the present study can arise from different methodologies. In the current study, anterior teeth were used and they were widened by mechanical instruments (post drill) and irrigated with high pressure and continuous up and down movements of syringe. Therefore, it seems that statistically insignificant differences among groups irrigated with 5.25% NaOCl, 17%EDTA and 17% EDTA + 2% CHX is because of mechanical pressure during irrigation. But, the chemical properties of irrigants are also remaining so these irrigants have better efficacy than control group. 

Our findings showed that there was no significant difference when different parts of post space surfaces were compared within one experimental group except 17% EDTA + 2% CHX which showed more remnants of smear layer in the apical third compared to coronal and middle thirds. This is in agreement with Elnaghy *et al. *[4]. No significant difference was found among all the root canal surfaces in EDTA in the study by Gu *et al. *[12], as well.

A number of studies explained that the larger diameter of canal in coronal and middle thirds contains more volume and also, the apical sclerosed dentine must not be overlooked [11]. But in the current study as it was mentioned, the canal diameter was as wide as it could be irrigated desirable at apical third.

Finally, it seems that using irrigation solutions especially 17% EDTA + 5.25% NaOCl have been effective in smear layer removal, although the mechanical pressure of saline was somewhat able to remove smear layer, as well. CHX seems to be ineffective on smear layer removal but according to its antimicrobial activity and its effect on preservation of dentine quality [2], it seems necessary to study the effect of this irrigant on bond strength in post-dentine interface. Other studies can be performed to assess the effect of other irrigants used here on desirable hybrid layer formation for bonding to post and measuring the bond strength of fiber posts to root canal dentine in a long period of time.

## Conclusion

Irrigating the post space using 17% EDTA followed by 5.25% NaOCl can effectively remove the smear layer. Our findings showed that 17% EDTA, 17% EDTA + 2% CHX and 5.25% NaOCl could not remove smear layer as effectively.
